# Relationship between stuttering severity in children and their mothers’ speaking rate

**DOI:** 10.1590/S1516-31802008000100006

**Published:** 2008-01-03

**Authors:** Ali Dehqan, Mehdi Bakhtiar, Sadegh Seif Panahi, Hassan Ashayeri

**Keywords:** Speech, Language, Communication, Stuttering, Mother(s), Fala, Linguagem, Comunicação, Gagueira, Mães

## Abstract

**CONTEXT AND OBJECTIVE:**

Stuttering is a complex disease that influences occupational, social, academic and emotional achievements. The aim of this study was to correlate the stuttering severity index with speaking rates of mothers and children.

**DESIGN AND SETTING:**

Cross-sectional study, at the child rehabilitation clinics of Tehran city.

**METHODS:**

35 pairs of mothers and their children who stuttered were studied. There were 29 boys and six girls, of mean age 8.5 years (range: 5.1-12.0). Speech samples from the mother-child pairs were audiotaped for approximately 15 minutes, until a reciprocal verbal interaction had been obtained. This sample was then analyzed in accordance with a stuttering severity index test and speaking rate parameters.

**RESULTS:**

The research results outlined a significant relationship between the mothers’ speaking rate and their children's stuttering severity.

**CONCLUSION:**

The results suggest that the mothers’ speaking rate should be incorporated in the assessment and treatment of stuttering.

## RESUMO

**CONTEXTO E OBJETIVO:**

Gagueira é uma doença complexa que tem influência nas realizações ocupacionais, sociais, acadêmicas e emocionais. A finalidade deste trabalho foi correlacionar o índice de gravidade da gagueira de crianças com as taxas de velocidade de fala das mães.

**TIPO DE ESTUDO E LOCAL:**

Estudo transversal, realizado na clínica infantil de reabilitação da cidade de Teerã.

**MÉTODOS:**

Uma amostra da fala de 35 crianças gagas pareadas com suas mães, sendo 29 meninos e 6 meninas, de 5:1-12:0 anos (idade média de 8.5), foi gravada por aproximadamente 15 minutos para avaliar a interação verbal recíproca, sendo então analisada de acordo com o índice de gravidade da gagueira e com os parâmetros de taxa de velocidade de fala.

**RESULTADOS:**

Os resultados da pesquisa mostraram uma relação significante entre a taxa de velocidade de fala da mãe e a gravidade da gagueira da criança.

**CONCLUSÃO:**

Os resultados sugerem que a taxa de velocidade da fala entre a mãe e a criança que gagueja deveria ser incorporada na avaliação e tratamento da gagueira.

## INTRODUCTION

Stuttering is described as a disorder of fluency and is characterized by part-word, whole-word and phrase repetitions, interjections, pauses and prolongations.^1^ Perhaps no speech problem has received more attention than stuttering. A wide variety of theories have been proposed based on the enormous volume of research findings. Some theories have proposed physiological factors for the onset of stuttering, such as bilateral hemispheric dominance,^2^ right hemispheric dominance for speech,^2,3^ brain damage,^4,5^ neuropsychological or neuromuscular dysfunction,^6^ laryngeal dysfunction^7^ and central auditory dysfunction.^8^ Data on the frequency of stuttering among relatives of those who stutter have led some investigators to propose a genetic component to stuttering.^9–13^ Others have suggested environmental factors for both the onset and the maintenance of stuttering, such as communicative stress,^14^ anxiety,^15^ personality and negative parent-child interactions.^16–18^

Although the recent advances in imaging techniques have shifted attention to neurological and/or physiological factors for the onset or cause of stuttering,^19,20^ the communication environment that stutterers live in may contribute towards maintaining the stuttering. Moreover, this communication environment that stuttering children live in may even play an important role in the success or failure of speech therapy. In other words, the role of the environment and, in particular, the linguistic and paralinguistic behavior and attitudes of parents have frequently been cited in both theoretical and clinical literature as presenting important correlations with the onset and development of stuttering among young children.^21–25^

Clinical intervention strategies currently used for children who stutter also frequently focus on the parents’ role, instructing them to alter their linguistic behavior (e.g. by reducing negative statements regarding their child's speech and/or stuttering) and their paralinguistic behavior (e.g. by reducing their overall speech rate).^24,26–37^ For example, Guitar and Marchinkoski^38^ and others^39^ reported that reductions in mothers’ speaking rates resulted in similar reductions in children's speaking rates and corresponding improvements in speech fluency for some children who stuttered.

Past studies have observed parents from a unidirectional perspective. Research questions have centered on the idea that the parents of stutterers were different from the parents of nonstutterers. To conduct bidirectional research, Meyers and Freeman^40^ explored the notion that the parents of children who stutter are “habitually fast talkers” and reported that the mothers of children who stutter spoke significantly faster than the mothers of nonstutterers did. Based on samples of the 15 longest perceptibly fluent utterances produced by each child, Meyers and Freeman^40^ also found that the stuttering children spoke significantly more slowly during their fluent speech than did their nonstuttering peers, and that the children with severe stuttering talked more slowly than did the children with moderately severe stuttering.^40^

It has been hypothesized that alterations in parental speaking rates may influence the speaking rates of children who stutter.^21,22,40,41^ Guitar and Marchinkoski^38^ investigated the effects on children's speech rate when their mothers talked more slowly and reported that when mothers substantially decreased their speech rates in a controlled situation, their children also decreased their speech rates.^38^

## OBJECTIVE

The aim of this study was to correlate the stuttering severity index with the speaking rates of mothers and their children who stutter.

## METHODS

**Subjects:** 35 pairs of mothers and their children who stuttered took part in this study. There were 29 boys and 6 girls, and their mean age was 8.5 years (range 5.1-12.0 years). The participants in this study were selected from the child rehabilitation clinics of Tehran University of Medical Sciences, in the city of Tehran. The selection criterion for the mothers of these children was that they should have normal speech, as diagnosed by a speech-language pathologist. The criterion for the children was that none of them had been evaluated for any fluency disorder or had received stuttering therapy prior to this study. Children who exhibited any clinically significant hearing, articulation, voice, language, psychological/emotional or physical problems that were considered atypical for their chronological age and level of development were excluded from this study.

Following data collection, a further assessment of stuttering was conducted by two certified speech-language pathologists. Children were classified as stutterers if they exhibited three or more within-word dysfluencies (i.e. sound prolongations, sound/syllable repetitions, monosyllabic whole-word repetitions or broken words) per 100 words during a 300-word sample of audiotaped conversation with their mothers, and if people in their environments had expressed concern regarding their speech fluency.

**Procedure:** Each child and his/her mother were seated facing each other at a small table on which a series of action pictures had been placed. They were instructed to talk about these action pictures. Each mother-child pair was audiotaped for approximately 15 minutes, or until a sufficient sample from the child was obtained. All mother-child verbal interactions were audiotaped.

Following the recording session, the utterances produced by each mother-child pair, i.e. the “unit(s) of language preceded and followed by a perceived pause or terminated by some change in inflection (rising or falling intonation)”,^42^ were orthographically transcribed by the first author. A 10-minute warm-up period of audiotaping was conducted prior to data collection, during which the subjects had the opportunity to become familiar with the equipment and materials present in the test environment. The number of syllables per utterance spoken by the mother and child was included on the transcript. The location and type of each within-word and between-word (i.e. multisyllabic whole-word repetition, phrase repetition, revision or interjection) speech dysfluency produced by each mother and child was also indicated. Finally, two examiners checked the transcriptions.

The speaking rate in the present study was equivalent to articulatory rate, which is defined as a number of syllables (AR-S) produced per minute of nonstuttered speech.^41,43^

The stuttering severity was determined by means of an international instrument (the Stuttering Severity instrument). This test assesses the frequency and duration of speech disruptions, and also the presence of physical concomitants associated with these disruptions.^44^ The children who stuttered were divided into three groups based on their stuttering severity: 12 subjects were in the mild group, 15 in the moderate group and eight in the severe group.

**Data analysis:** One-way analysis of variance (ANOVA) was performed to compare the mothers’ speaking rates and their children's stuttering severity and also to compare the children's speaking rates and their stuttering severity. The Pearson correlation was used to compare the speaking rates of the mothers and their stuttering children. The SPSS software (version 12) was used for data analysis.

## RESULTS

**Figure 1:**
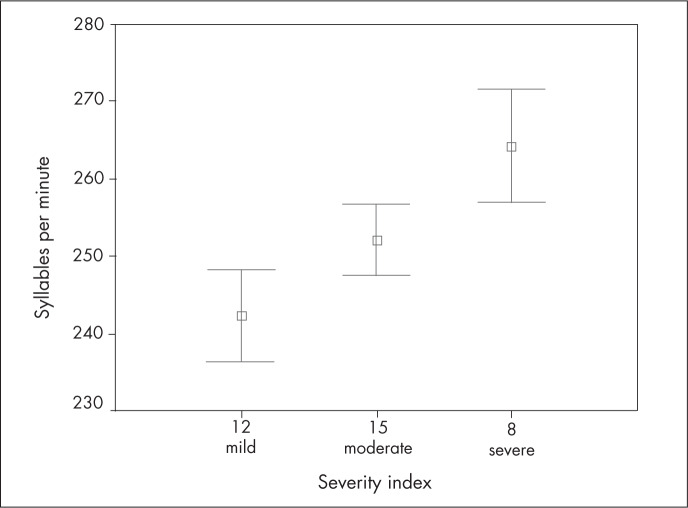
Severity index and syllables per minute among mothers.

As illustrated in Table 1 and Figure 1, there was a significant relationship between the means for the mothers’ speaking rates and their children's stuttering severity. In other words, faster speaking rates among mothers were associated with greater stuttering severity in their children (p < 0.01).

**Table 1.** Comparison between mothers’ speaking rates and children's stuttering severity

Children's stuttering severity	Mothers’ speaking rate		95% CI		F	p-value
	Mean	SD	Lower limit	Upper limit		
**Mild** (n = 12)	242.30	2.96	240.42	244.19		
**Moderate** (n = 15)	252.08	2.32	250.80	253.37	137.97	< 0.01*
**Severe** (n = 8)	264.18	3.68	261.10	267.26		

* statistically significant; CI = confidence interval; SD = Standard Deviation.

On the other hand, as shown in Table 2, there was a significant negative correlation between the means for the mothers’ speaking rates and their children's speaking rates (p < 0.01). Thus, the faster the mother's speaking rate was, the slower her child's speaking rate was.

**Table 2.** Comparison between speaking rates of mothers and children

	Speaking rate		Pearson correlation	p-value
	Mean	SD		
**Mother**	251.49	8.70	- 0.92	< 0.01*
**Child**	115.50	17.70		

* statistically significant; SD = standard deviation.

**Figure 2:**
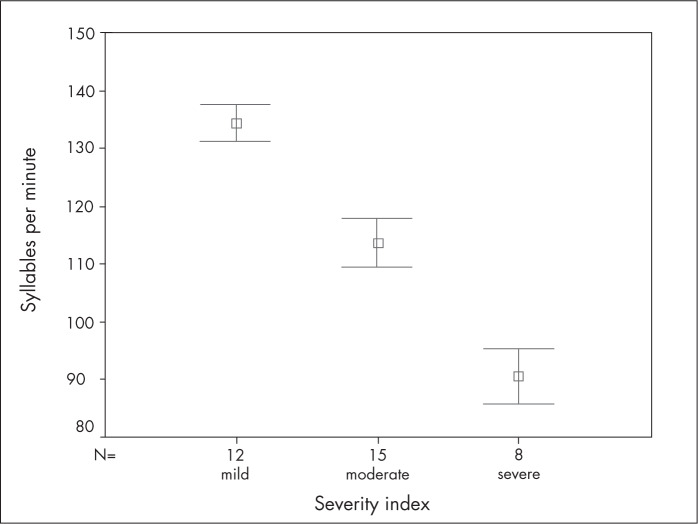
Severity index and syllables per minute among the children who stuttered.

Finally, Table 3 and Figure 2 present an intragroup comparison between the children's speaking rates and their stuttering severity. This showed a significant correlation (p < 0.01) such that increased stuttering severity was associated with decreased speaking rates among the children who stuttered. In other words, the children with severe stuttering severity had a slower speaking rate than the moderate group did, and the moderate group had a slower speaking rate than the mild group did.

**Table 3.** Comparison between children's speaking rates and their stuttering severity

Children's stuttering severity	Children's speaking rates		95% CI		F	p-value
	Mean	SD	Lower limit	Upper limit		
**Mild** (n = 12)	134.45	5.07	131.22	137.67		
**Moderate** (n = 15)	113.66	7.55	109.48	117.85	137.97	< 0.01*
**Severe** (n = 8)	90.54	5.72	85.75	95.33		

* statistically significant; CI = confidence interval; SD = standard deviation.

## DISCUSSION

Despite the behavioral complexity of a stuttering problem, dysfluency often plays a primary role in differential diagnostic decisions and treatment evaluations. It is known that absolute continuity of speech production is physiologically impossible. A perception of continuous speech can be obtained from the number of audible speech utterances and the shortness of the physiological pauses (e.g. intervals for swallowing and breathing), and from the linguistic pauses (e.g. memory effects and lexical access) that are pertinent and expected from any speaker.^45^ The present study regarded the mother's speaking model as an important part of her child's interaction environment, which had an impact on the child's speaking model and was associated with the severity of the problem. This basic result held true in the present study, such that with increased speaking rates among the mothers with stuttering children, their children's stuttering severity would also be increased.

As was noted in the present study, the mothers with high speaking rates imposed more time pressure and communication stress on their small conversation partners. Thus, their children felt under more stress, which would result in enhancement of their stuttering severity.

On the other hand, enhancement of the children's stuttering severity would lead their mothers to get into a “nervous state” and they would compensate for this by increasing their speaking rates, in the hope that their children might increase their speaking rates.

Another result obtained from the present study was that, with increasing stuttering severity among these children, their speaking rate decreased. This result is also in line with the findings of Meyers and Freeman.^40^ Furthermore, the results from de Andrade et al.^45^ and from the present study have confirmed the findings previously published regarding speakers of American English, thereby pointing towards a direct relationship between increases in the stuttering severity index and reductions in speech rate.^45^

The latter result, showing that there is an interesting negative correlation between mother's and children's speaking rates, is in line with the findings of Ainsworth and Fraser,^21^ Conture and Fraser,^22^ Costello^41^ and Meyers and Freeman.^40^ All of these other studies hypothesized that alterations in parental speaking rates influenced the speaking rates of stuttering children. Moreover, the present study was in line with Meyers and Freeman,^40^ in concluding that the more the child stuttered, the slower he talked, and the slower the child talked, the faster the mother interacting with him talked. However, it is equally possible to interpret this analysis as demonstrating that the faster a mother spoke, the more the child stuttered, and the more he stuttered the slower he talked, and so forth.

There are several possible reasons why mothers might use a faster speaking rate when talking to a slow-talking or stuttering child. First, stuttering behavior may alter dialogue patterns. That is, a slow-speaking and/or stuttering child may disrupt the pace of the ongoing interaction, thus prompting the mother to speed her rate in the hope of increasing the child's rate. By talking faster, a mother may press the child to talk faster, and talking faster may lead to increased stuttering. Alternatively, a child's struggle for fluency, or his frequent dysfluency, may create internal discomfort, tension, anxiety or “nervousness” in his mother. Such internal reactions may alter a mother's speech motor behavior, thereby causing her to speak faster.^40^

## CONCLUSION

The findings from the present study suggest that the speech rates of mothers and their children who stutter are important indicators of fluency levels among stuttering children and should be incorporated in the assessment and treatment of stuttering. Mothers, as their children's main communication partners, have an important role in the therapy process and in setting clinical strategies.
